# Study Protocol for a Randomized Controlled Trial of Choral Singing Intervention to Prevent Cognitive Decline in At-Risk Older Adults Living in the Community

**DOI:** 10.3389/fnagi.2018.00195

**Published:** 2018-07-10

**Authors:** Jasmine Tan, F. H. Maurine Tsakok, Elisabeth K. Ow, Bernard Lanskey, Kian Siong Darius Lim, Lee Gan Goh, Chay-Hoon Tan, Irwin Kee-Mun Cheah, Anis Larbi, Roger Foo, Marie Loh, Caroline Kai Yun Wong, John Suckling, Jialiang Li, Rathi Mahendran, Ee-Heok Kua, Lei Feng

**Affiliations:** ^1^Department of Psychology, Goldsmiths, University of London, London, United Kingdom; ^2^Maurine Tsakok Inc., Singapore, Singapore; ^3^Department of Psychological Medicine, Yong Loo Lin School of Medicine, National University of Singapore, Singapore, Singapore; ^4^Yong Siew Toh Conservatory of Music, National University of Singapore, Singapore, Singapore; ^5^Darius Music, Singapore, Singapore; ^6^Division of Family Medicine, Yong Loo Lin School of Medicine, National University of Singapore, Singapore, Singapore; ^7^Department of Pharmacology, Yong Loo Lin School of Medicine, National University of Singapore, Singapore, Singapore; ^8^Department of Biochemistry, Yong Loo Lin School of Medicine, National University of Singapore, Singapore, Singapore; ^9^Singapore Immunology Network (SIgN), Agency for Science, Technology and Research (A*STAR), Singapore, Singapore; ^10^Genome Institue of Singapore (GIS), Agency for Science, Technology and Research (A*STAR), Singapore, Singapore; ^11^Translational Laboratory in Genetic Medicine, Agency for Science, Technology and Research (A*STAR), Singapore, Singapore; ^12^Clinical Imaging Research Centre (CIRC), Singapore, Singapore; ^13^Department of Psychiatry, University of Cambridge and Cambridge and Peterborough NHS Foundation Trust, Cambridge, United Kingdom; ^14^Department of Statistics & Applied Probability, Faculty of Science, National University of Singapore, Singapore, Singapore

**Keywords:** singing, cognitive decline, RCT, dementia prevention, neuroplasticity

## Abstract

**Introduction**: This study is a parallel-arm randomized controlled trial evaluating choral singing’s efficacy and underlying mechanisms in preventing cognitive decline in at-risk older participants.

**Methods**: Three-hundred and sixty community-dwelling, non-demented older participants are recruited for a 2-year intervention. Inclusion criteria are self-reported cognitive complaints, early cognitive impairment based on neuropsychological test scores or multiple risk factors of dementia. Participants are randomized to either weekly choral singing sessions or general health education. The primary outcome is cognitive performance, measured by a composite cognitive test score (CCTS). Secondary outcomes include depression, anxiety and neuropsychiatric symptoms; perceived stress; sleep quality and severity of dementia symptoms. Underlying mechanisms are examined using blood- and urine-based biomarkers and neuroimaging.

**Results**: Screening began in July 2016. The first group of participants (*n* = 93) have been recruited. Intervention and control treatments are ongoing and will end in December 2019.

**Discussion**: An evidence-based singing intervention for dementia prevention holds potential for healthcare savings and societal welfare.

**Trial Registration**: NCT02919748, IRB Approval Number: NUS 2508.

## Introduction

Cognitive function declines with advancing age and the prevalence and incidence of dementia rises dramatically in later life. Impaired cognitive function presents a major obstacle to active aging, as it limits the ability to work, live and socialize, contributing to functional disability. To this end, we propose choral singing as a novel approach towards the prevention of cognitive decline and dementia.

Extensive research has gone into drug-based methods of preventing cognitive decline (Bateman et al., 2017), but non-pharmacologic methods are also promising. Participation in various cognitive, social and productive activities improves cognitive function and lowers risk of dementia (Feng et al., 2011), and this increase in social engagment particularly benefits single and widowed elderly (Feng et al., 2014). A study among elderly Chinese Singaporeans found significant reduction in depression, a risk factor for dementia, following psychosocial group activity interventions such as mindfulness therapy, music reminiscence therapy, art therapy and tai-chi (Kua et al., 2013).

Cognitive training has been demonstrated to be effective in improving cognitive function or delaying cognitive decline in the elderly. Encouragingly, functional gains from cognitive training have been reported to last up to 5 years (Willis et al., 2006; Valenzuela and Sachdev, 2009). In Singapore, a brain-computer interface-based cognitive training showed promise in improving memory and attention in healthy older people (Lee et al., 2013). However, more cost-effective and engaging methods are needed.

There is a growing body of research on the role of choral singing in health promotion and psychological well-being. Qualitative and descriptive studies reveal that the perceived benefits of choral singing include social connection, emotional and psychological well-being, improved physical health, and improved concentration and memory (Skingley et al., 2015; Hallam and Creech, 2016). These observations have been tested empirically with varying levels of rigour. Interventional studies on choral singing initiatives for community-dwelling elderly have found improved morale and decreased loneliness (Cohen et al., 2006) as well as improved mental health-related quality of life (QOL), and reduced levels of anxiety and depression compared to controls (Coulton et al., 2015). Choral singers also report higher physical health-related QOL than matched controls (Johnson et al., 2017). Older adults in a choral singing intervention not only had higher self-ratings of health, but also better objective health outcomes such as fewer visits to the doctor (Cohen et al., 2006). Salivary immunoglobulin A, a marker of immune system strength, increases significantly after singing, suggesting that singing activates the immune system (Beck et al., 2000; Kreutz et al., 2005).

Standardized measures quantifying cognitive improvements have only been utilized in recent years. Singing programs have been found to improve cognition in individuals with dementia, improving scores on the MMSE and the Clock Drawing Test (Maguire et al., 2015). A small pilot trial found improved psychomotor processing speed and reduced neuropsychiatric symptoms of dementia after 6 months of weekly singing sessions (Satoh et al., 2015). An RCT involving 89 dementia patient-caregiver dyads demonstrated that 10 weeks of singing significantly improved mood, orientation, and remote episodic memory, and to a lesser extent, attention, executive function and general cognition (Särkämö et al., 2014). However, singing’s effect on cognition has not been examined in community-living, non-demented elderly.

**Figure 1:**
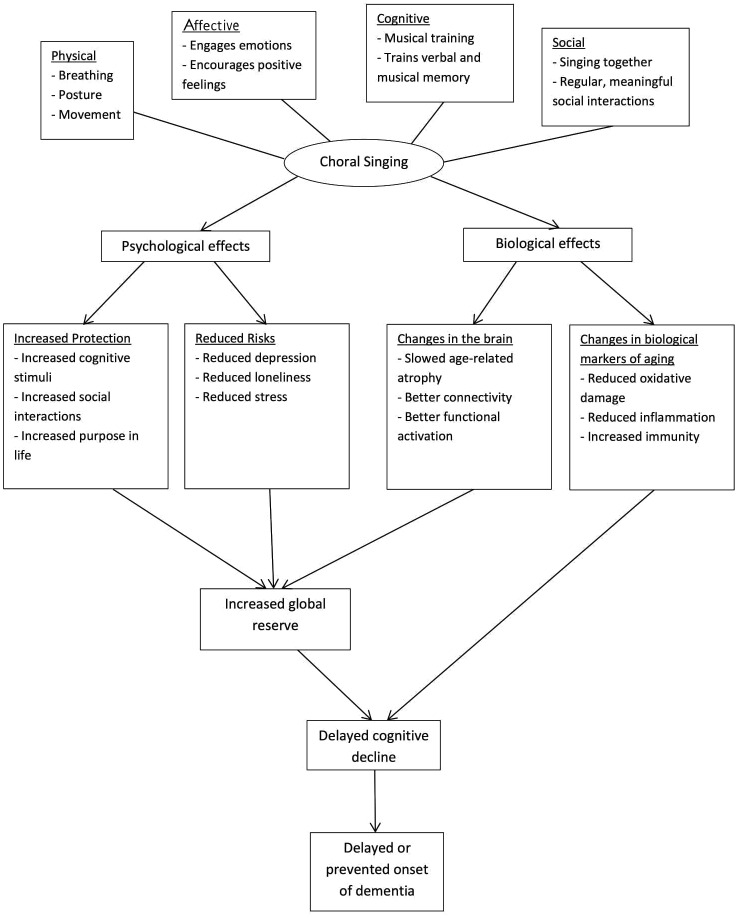
Theoretical model of choral singing’s efficacy in slowing cognitive decline and preventing dementia. Choral singing is hypothesized to delay cognitive decline and prevent dementia by increasing protection of the brain and reducing the risk burdens of dementia. As a complex activity, its effects can be attributed to the cognitive, social, emotional and physical aspects of choral singing. The biological mechanisms behind its effects may be observed from changes in brain structure and function and markers of biological aging.

Choral singing is a cost-effective activity (Coulton et al., 2015) which involves physical, cognitive, social and affective components. The multi-dimensionality of this activity may confer greater health benefits over activities involving only one component, and the social aspect of this activity can have a stronger protective effect compared to activities which can be pursued individually (Karp et al., 2006). The benefits may also be compounded as participants remain involved for a long time, given the intrinsic appeal of singing (Cohen et al., 2006). Choral singing may delay cognitive decline and prevent dementia through conferring protection and reducing risks of dementia (see Figure 1).

Existing research on the benefits of singing have their limitations (Clark and Harding, 2012). Many trials use empty control or “usual care” as the comparison group, a flawed design as generic components of group activities may contribute to observed outcome changes. To better identify the specific efficacy of choral singing, this study includes an active control arm, a health education program. A comprehensive set of measures covering the psychiatric, cognitive and mental symptoms of dementia allows careful tracking of all possible effects of the intervention.

The neuroplasticity of the brain forms the scientific basis for the potential effectiveness of the intervention. Interventions have been found to affect neural markers such as cortical thickness (Staubo et al., 2017), white matter integrity (Engvig et al., 2012) and functional activity (Hosseini et al., 2014). The neuroimaging evidence associated with cognitive improvement points towards a slowing or even reversal of age-related atrophy, the extent of change correlating with training-related cognitive improvements (Engvig et al., 2010, 2012). In addition, the neuroplasticity induced by long-term musical training is well-established. Music-making utilizes both sensory and motor systems, and involves numerous higher-order cognitive processes, resulting in widespread alterations to the brain (Herholz and Zatorre, 2012). Indeed, the only study to date examining neural changes after a singing intervention reported a reduction in functional MRI activity during singing attributable to practice-related reorganization (Satoh et al., 2015). Use of neuroimaging and biomarkers can provide a more detailed assessment of the pathological processes of cognitive decline and reveal how the intervention can influence them. Neuroimaging data on intervention-related changes in volume, structural integrity and functional activation can provide important insights into the nature and duration of an intervention’s effect beyond behavioral observations (Lustig et al., 2009). Measuring changes in markers of biological aging will shed light on relevant pathways that may mediate choral intervention and the hypothesized efficacy. For this purpose, we have carefully selected biomarkers of immune function, inflammation and oxidative damage.

The study’s main objective is to evaluate the clinical efficacy and elucidate possible underlying mechanisms of choral singing in dementia prevention through an RCT. We hypothesize that choral singing can prevent cognitive decline among community-dwelling elderly at high risk of dementia. The primary outcome is cognitive performance, measured using a composite cognitive test score (CCTS). The underlying mechanism involving changes in brain structure and function will be probed using MRI. Changes in cognitive outcomes will be correlated to a panel of carefully-selected blood- and urine-based biomarkers. Secondary outcomes include measures of mental health and sleep quality.

## Materials and Methods

### Participants

Trial participants are community-living elderly aged 60 years and above. Some are participants from two previous studies on healthy aging (approval number: NUS 1351 and NUS 2501) who had consented to be contacted for future research, with additional participants recruited through door-to-door visits. Screening is done by trained research nurses and psychologists. Participants are included if they presented self-reported subjective cognitive complaints, objective cognitive impairment based on neuropsychological test scores (age- and education-standardized Z-scores in the range of 0 to −1.5 for at least five tests, out of 10) or at least two risk factors of dementia (family history of dementia, physical inactivity, low social engagement, heart disease, transient ischemia attack, diabetes, head injury, living alone and depressive symptoms), but are not demented (Clinical Dementia Rating global score = 0). Exclusion criteria are conditions preventing effective engagement in the intervention, such as: terminal illness, stroke, aphasia and marked hearing impairment, as well as participation in another interventional study.

Eligible participants are given sufficient information about the study and provide their informed consent prior to a screening assessment. Results of the screening also act as a baseline measurement. Recruitment will proceed until a total of 360 participants is reached.

### Randomization

Following informed consent, randomization into intervention or control arm is performed with a 1:1 allocation ratio. Balanced treatment assignments will be achieved using permuted block randomization. The block length is determined by an independent statistician and is not made known to the clinical investigators or site personnel. Opaque, sequentially numbered and sealed envelopes are prepared for the randomization process. Authorized site personnel open the randomization envelopes in sequential order to determine the participant’s treatment arm and the identification number to be used for that participant.

Participants randomized to the intervention arm participate in choral singing for 2 years while participants in the control arm take part in general health education and group activities.

#### Intervention: Choral Singing

The intervention protocol was designed by a professional choir conductor (KSDL) with input from a senior academic musician (BL), an experienced choral singer (FHMT) and choral conductors experienced in working with older adult choirs. It aims to provide a full choral experience pitched at the level of an older adult group with no formal musical background, teaching the fundamental aspects of choral singing and singing in an ensemble in an energetic and positive manner. The philosophy of this approach is to focus on the joy of choral singing and to provide a good social environment. While the protocol here focuses primarily on rehearsal principles, a regular schedule of public performances in a variety of semi-formal community settings (approximately four times a year) is also scheduled to make the experience as authentic as possible.

The choral singing program is held weekly for 2 years. Each session is 1 h long. Each choral session incorporates the social, physical and musical aspects of choral singing. Members of the choir are given time to socialize before and after rehearsal. Rehearsals begin with 15 min of physical exercises (adapted for the age and physical mobility of participants) and vocal warmups. Physical aspects of choral singing are discussed in exercises to promote healthy posture for singers, vocal and body awareness and the understanding of good effective breathing for singers. The focus during choral sessions is to educate singers to understand the concept of sound, the mechanics of the singing voice and to be able to differentiate good from bad singing. The participants are then taught more in-depth skills of correct, healthy vocal production using good breathing techniques, support and listening skills. Later in the program, participants learn to sing in different parts. The parts are taught aurally and slowly, helping each singer to understand that they represent different lines in the musical harmony at any point in two- or more-part harmony. Choir members will work on listening to and synchronizing with each other.

At the end of every rehearsal, social interaction is incorporated as choir members are encouraged to perform for each other and opportunities are created for enthusiastic singers to perform. A consistent team of choral conductors will prepare repertoire and conduct rehearsals. The weekly progress of participants is tracked throughout the intervention to better enable the conductor to develop their vocal potential.

The protocol is described in a written manual so it is easily transferable and modifiable for the standards of music providers in other groups if this interventional initiative is scaled up. The choir training manual will be made available to researchers and practitioners after the completion of this RCT. Requests can be addressed to LF.

#### Control: Health Education and Group Activities

Sessions consist of short talks on a health-related topic followed by group activities that require memory work and the acquisition of certain skills, but do not involve singing. Activities are comparable with the intervention group in terms of duration and intensity of social interaction and intellectual stimulation. The frequency of the sessions is matched exactly with the choral singing sessions (weekly 1 h sessions for 2 years).

The topic and content of the group activities are designed by study investigator LGG and conducted by him and research nurses employed under the project. Physical activity is incorporated with 10 min of exercise at the end of every session. Topics covered include attention to lifestyle, managing high blood pressure and diabetes, healthy eating for seniors, the prevention of falls, and the handling of anxiety, depression and dementia.

For both intervention and control arms, participant attendance is taken at each session and monitored closely each week. The research team contact absent participants to encourage them to continue coming for the intervention and control activities. If participants fail to attend, their reasons are recorded.

### Ethics Statement

This study was carried out in accordance with the recommendations of the National University of Singapore (NUS) with written informed consent from all subjects. All subjects gave written informed consent in accordance with the NUS Human Biomedical Research guidelines. The protocol was approved by the Institutional Review Board of the National University of Singapore (Approval Number: NUS 2508).

### Assessments and Outcome Measures

A comprehensive assessment protocol with cognitive and psychological measures, blood and urine biomarkers and brain MRI measures has been designed for this study. Participants undergo questionnaire-based interviews, cognitive testing, clinical assessments, specimen collection and MRI prior to, during and at the end of the intervention (Table 1). Assessors at each follow-up are blind to group allocation.

**Table 1:** Measurements and visit schedules.

	Measurement	Baseline	6 months	1 year	2 year
1	Social, demographic and lifestyle data	√			
2	Medical conditions and medications	√			
3	Mini mental state examination (MMSE)	√	√	√	√
4	Subjective memory and cognitive complaint	√		√	√
5	Neuropsychological testing/assessment	√		√	√
6	Clinical dementia rating (CDR)	√		√	√
7	Neuropsychiatric inventory (NPI)	√		√	√
8	Activities of daily living (ADL)	√		√	√
9	Instrumental activities of daily living (IADL)	√		√	√
10	Pittsburgh sleep quality index (PSQI)	√	√	√	√
11	Geriatric depression scale (GDS-15)	√	√	√	√
12	Geriatric anxiety inventory (GAI)	√	√	√	√
13	Perceived stress scale (PSS)	√	√	√	√
14	Rhythm experiment	√		√	√
15	The social cognition and behavior assessment	√		√	√
16	Gold-MSI	√			√
17	Venous blood sample collection	√		√	√
18	Urine sample collection	√	√	√	√
19	Brain MRI, blood pressures, pulse rates	√		√	√

#### Cognitive and Psychological Measures

The *primary outcome* of the study is change in cognitive performance as measured with a CCTS based on results from a neuropsychological test battery. The CCTS is the average of Z-scores standardized to the baseline mean and standard deviation of the trial participants, with higher scores representing better cognitive performance. The test battery evaluates cognitive function in specific cognitive domains such as verbal memory (Rey Auditory Verbal Learning Test; Rey, 1964), attention (Digit Span (WAIS-III UK); Wechsler, 1997), executive function (Color Trails Test 1&2; D’Elia et al., 1996), visuospatial functioning (Block Design (WAIS-III UK); Wechsler, 1997), information processing speed (Symbol-Digits Modalities Test; Smith, 2013) and language (Boston Naming Test; Kaplan et al., 1983). Trained research staff administer the tests in the participant’s most proficient language. The effects of language and culture are minimized by using test items familiar to the study population.

*Secondary cognitive outcomes* include cognition measured by a modified version of the Mini-Mental State Examination (MMSE; Folstein et al., 1975; Feng et al., 2012), the Montreal Cognitive Assessment (MoCA; Nasreddine et al., 2005) and scores from each of the above neuropsychological tests. Individual test scores are standardized (z-transformed) using normative data of the local population (see Supplementary Tables S1, S2).

Other secondary outcomes measured are severity of dementia symptoms (Clinical Dementia Rating scale; Morris, 1993); neuropsychiatric symptoms (Neuropsychiatric Inventory; Cummings et al., 1994); depressive symptoms (Geriatric Depression Scale; Yesavage and Sheikh, 1986); anxiety symptoms (Geriatric Anxiety Inventory (GAI); Pachana et al., 2007); perceived stress (Perceived Stress Scale (PSS); Cohen et al., 1983); and sleep quality (Pittsburgh Sleep Quality Index (PSQI); Buysse et al., 1989).

We also collect data on musical skill and engagement (Goldsmiths Musical Sophistication Index; Müllensiefen et al., 2014), social cognition and rhythmic entrainment but these measures will not be used as trial outcomes for primary analysis.

#### Biomarkers

Collection of venous blood samples and urine is carried out by qualified research nurses to track markers of biological aging. From Peripheral Blood Monocytes (PBMCs), immune profiling is done using classical markers of senescence (CD57, KLRG1) and exhaustion (PD1) of T cells and identification of subpopulations of B cells and myeloid cells with a focus on pro-inflammatory cells. Measuring proliferation profile and pro-inflammatory cytokines will allow us to assess the functional capacity of the cells.

LC-MS/MS will be used to measure markers of oxidative damage (F2-isoprostanes; F4-neuroprostanes; 8-hydroxy-2′-deoxyguanosine; allantoin and urate concentrations; hydroxyeicosatetraenoic acids) in plasma and urine. Protein carbonyl contents in the plasma are analyzed using a commercial assay kit. Genomic DNA is extracted from PBMCs using QIAamp DNA mini kit (Qiagen, Germany). Telomere restriction fragment (TRF) length analysis by southern blot is used to estimate average leukocyte telomere length (TL; TeloTTAGG TL assay, Roche Diagnostics). Briefly, restriction digestion of 1 μg of DNA is carried out using Hinf I/Rsa I enzymes at 37°C for 2 h. The digested product is resolved on 0.8% agarose gel and then transferred onto a nylon membrane (Hybond, N+ Amersham, UK) overnight. Hybridization is performed at 42°C with digoxigenin labeled telomeric probe and the TRF smear is detected using a digoxigenin luminescent detection procedure. The smear signal is recorded on X-ray films which are then digitized. Average TL is estimated by comparison to a 1 kb plus DNA ladder. TL estimation of samples is done using TeloTool software (Matlab Central, Austria), which permits an accurate estimation of TL.

The levels of stable biomarkers of oxidative damage will give an indication of the overall oxidative state of the individual. As psychological stress and cognitive dysfunction are associated with increased oxidative damage, we hypothesize that the levels of these peripheral oxidative damage biomarkers will decrease in the intervention group relative to baseline and the control group. We postulate that the intervention would affect the senescence profile of immune cells from participants including their secretory profile (cytokine measurement and PD1 expression) and the replicative senescent status (proliferation and CD57 expression). Inflammatory monocytes (CD16+) were shown to be up-regulated in aging and in cases of inflammatory conditions. The impact of the intervention on the overall pro-inflammatory profile should be noticed in either the lymphoid or myeloid compartment.

Epigenome-wide methylation data will be generated for the samples using the state-of-the-art Illumina Infinium MethylationEPIC BeadChip (Pidsley et al., 2016). The array covers over 850,000 methylation sites per sample at single-CpG site resolution, providing unparalleled coverage of CpG islands, RefSeq genes, ENCODE open chromatin, ENCODE transcription factor binding sites, and FANTOM5 enhancers, as well as regions identified by the ENCODE project as potential enhancers.

DNA methylation will be quantified in the baseline DNA samples from venous blood. Bisulfite conversion of genomic DNA will be done using the EZ DNA Methylation kit, which requires ~500 ng of input DNA. The methylation profiling will be repeated for DNA samples from venous blood collected at the 2 year time point. We expect methylation age (calculated from the 353 CpG sites) to be lower in the participants assigned to the choral singing group relative to the control group as choral singing is expected to be associated with healthy aging.

#### MRI Assessment Battery

Participants are scanned with a 32-channel head coil on a 3T Prisma Siemens MR scanner at the Clinical Imaging Research Centre (CIRC), located at the National University of Singapore. Both structural and functional data are acquired during the MRI scan, with the full protocol detailed in Table 2.

**Table 2:** MRI scan timeline (sequences, durations, purposes).

	Sequence	Purpose	Time (min)
1	Localizer	For field-of-view positioning	0:14
2	3D MPRAGE	High-resolution anatomical scan	5:01
3	BOLD_Resting	Resting state functional connectivity	9:01
4	BOLD_FNPA	Brain activations under FNPA task	7:36
5	BOLD_SIN	Brain activations under singing task	2:36
6	BOLD_auditory localizer	Localization of auditory cortex	3:06
7	Gre field map	For distortion correction	1:07
8	Ep2d_diff (DTI)	White matter integrity	9:06
9	T2_SWI	For detection of micro-bleeds	4:54
10	T2_TSE_ FLAIR	To detect and characterize structural lesions	4:28
11	BOLD_TUNE	Brain activations under musical memory task	3:06
		TOTAL	50:15

The structural scans will allow the tracking of changes in brain volumes and rates of atrophy over time for the whole brain, hippocampus and temporal horn. In addition, changes in white matter integrity over time will be examined through fractional anisotropy, mean diffusivity, and radial diffusivity. The number of white matter lesions and cerebral micro-bleeds will also be recorded. If the choral-singing intervention does indeed confer greater protection from cognitive decline than general health education, a lower rate of structural change should be observed in participants assigned to the choral singing group as opposed to the control group.

Besides structural data, both task-based and functional data are collected as well. The resting state functional data will be analyzed for the strength of intra-network connectivity in large scale networks like the default mode network. The network connectivity of the choral singing group should be more coherent than that of the control group if the choral-singing intervention confers greater protection from cognitive decline than general health education. The task-based functional data are primarily exploratory in nature and are meant to see if participants in the choral singing group are able to fare better in tasks involving auditory stimuli. The tasks are described below.

##### Face-Name Paired Associates Task

The paired associates task will assess encoding and retrieval processes. The task consists of 10, 30-s encode blocks alternating with recall blocks, with each block separated by a resting baseline block. In the encoding blocks, participants are shown pictures of faces paired with audio recordings of names and asked to indicate if they feel the name fits the displayed face. This ensures that participants are attending to the stimuli and processing the face-name pairing, allowing them to carry out the recall task. In the recall blocks, participants are shown the same faces paired with audio recordings of names. Half of the names would have been previously paired with the same face, while the other half would not. Participants indicate if the face-name pairings were the same as before.

##### Singing Task

Participants listen to five short tunes and hum each melody after hearing it. The familiar melodies are taken from the repertoire of the choral intervention.

##### Melodic Memory Task

The task involves musical encoding and recall. In the encoding phase, participants are instructed to listen carefully to 20 short (4–6 s) newly-composed melodies. Stimuli will be presented in a block design, with five blocks of four melodies each, separated by resting baseline blocks. In the recall phase (taking place outside the scanner), participants hear 40 melodies and indicate which melodies were heard in the encoding phase. They are also asked to rate, on a scale of 1–7, how much they liked each melody, for the assessment of implicit memory.

These fMRI tasks were designed to involve the sense of hearing. Unfortunately, both memory functions and the auditory cortex are located in the temporal lobe. An auditory localizer scan allows exclusion of the auditory cortex when performing memory-related analyses.

#### Frequency of Assessments

Follow-up assessments will be conducted 6 months after the start of the intervention, at the end of the first year and at the end of the second year. Details of all measurements at baseline and each follow-up visit are listed in Table 2.

## Methods of Analysis

CCTS at each follow-up visit, absolute changes from baseline and percentage changes from baseline during the intervention period will be compared between the two arms using Student’s *t*-test and repeated-measures analysis of variance (ANOVA). Following this, a linear mixed-effects model will be conducted to examine the main effect of time and group allocation, and the interaction between time and group allocation, adjusting for potential confounders where necessary. Fisher’s exact tests will be used to compare the difference in dichotomous outcome measures such as cognitive decline, defined as the one or more points in MMSE score, between the two arms. Multiple logistic regressions with adjustment for important baseline characteristics will be conducted in the event of important baseline non-comparability. Statistical analysis will be conducted using SPSS, Stata or R. Interim analysis will be conducted for the first group of participants using data collected at 6 months, 12 months and 24 months, respectively.

The statistical analysis for DNA methylation will be performed using a modified version of the CPACOR pipeline to eliminate potential confounders such as bisulfite conversion, batch effect, and white blood cell composition (Lehne et al., 2015). Marker intensities will be normalized by quantile normalization. We will use the epigenome-wide methylation scores to impute white blood cell subsets using the Houseman method (Houseman et al., 2014). A principal components analysis will also be performed to quantify latent structure in the data, including batch effects. Single-marker tests using logistic regression will be used to examine the association of each autosomal CpG site with condition (choral singing/control), adjusted for age and sex. We will also calculate the methylation age using the 353 CpG sites previously identified (Horvath, 2013). Principal components from intensity values of Infinium control probes, which accounts for various technical parameters such as bisulfite conversion batch, will also be included as covariates in the regression models. Finally, the association results will be adjusted for genomic control inflation factor.

MRI images obtained from baseline and follow-up visits will be used to track relevant features such as hippocampal, whole-brain, total cerebral volume and annualized rate of atrophy; total ventricular volume, temporal horn volume; white matter volume and shape; fractional anisotropy and mean diffusivity and radial diffusivity; and the number of white matter lesions and cerebral micro-bleeds. Software such as FreeSurfer and BrainVoyager will be used in image processing and analysis. Functional connectivity analysis will be conducted using group ICA to track changes in networks such as the default mode network and the social brain network. As the face-name paired associates and melodic memory tasks are expected to involve episodic memory, the hippocampus is an *a priori* area of interest. Hemodynamic response function (HRF) time-locked to the presentation of stimuli will be extracted and analyzed.

For all data sets, a linear mixed-effects model will be used to analyze changes in mean responses over time for each group and the interaction term between group allocation and the time variable. A significant group × time interaction will indicate an effect of the choral singing intervention, following which appropriate *post hoc* tests can be done.

Intention-to-treat and per protocol analyses will be conducted. Compliance is monitored. Sensitivity analysis will be conducted by excluding subjects whose compliance (attendance) rate is below a specific cutoff point.

## Progress

**Figure 2:**
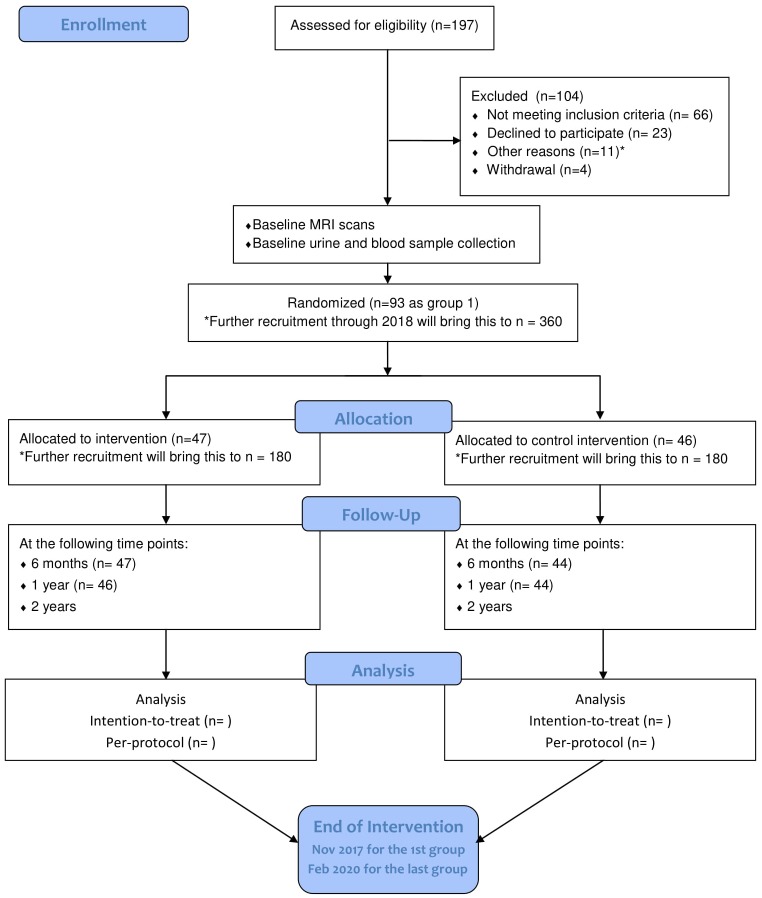
Flow diagram of participants’ progression through phases of RCT as of May 2017. Adapted from Schulz et al. (2010). *Other reasons: four with hearing impairment, one with visual impairment, one with advanced cancer, two participating in other studies, two received recent neuropsychological assessment, one relocated outside the defined recruitment area.

Starting from July 2015, 404 community-living seniors were invited to the screening. Of these, 197 participated and 93 were recruited. The target of identifying and randomizing the first group of participants was achieved in October 2015. The intervention was initialized on schedule with the choral singing intervention starting on 29 October 2015 and the control intervention starting on 4 November 2015. More participants will be recruited through 2017 and 2018 until the target of 360 is met. Final data collection is expected to take place in February 2020 (see Figure 2 for participant flowchart; Schulz et al., 2010). Baseline measurements, MRI scans, bio-sampling and follow-up measurements at 6 months and 1 year after the start of the intervention have been collected for the first batch of participants. Electronic data entry and processing are ongoing. The baseline characteristics of the first group of participants are summarized in Table 3.

**Table 3:** Baseline characteristics of the first 93 randomized trial participants.

Variable	Choral singing (*n* = 47)	Controls (*n* = 46)	*p*-value
Age (year), mean (SD)	70.98 (0.835)	69.39 (0.779)	0.168
Women, n (%)	76.6	80.4	0.652
Education, n (%)			0.235
No formal schooling	21.3	8.7	
Primary and below	53.2	63.1	
Secondary and above	25.5	28.2	
Marital status			0.925
Married	55.3	54.3	
Divorced/Widowed/Single	44.7	45.7	
Living alone, n (%)	14.9	15.2	0.965
SMCC total score, mean (SD)	39.32 (0.964)	38.41 (0.642)	0.438
MMSE total score, median (IQR)	29 (27–30)	29 (27–30)	0.720
MoCA total score, median (IQR)	26 (24–28)	26 (25–29)	0.766
GDS total score, median (IQR)	2 (0–3)	1 (0–2.25)	0.246
GAI total score, median (IQR)	0 (0–4)	0 (0–1)	0.122
PSS total score, mean (SD)	15.53 (0.633)	14.91 (0.493)	0.444
GoldMSI score, mean (SD)	58.94 (1.71)	55.93 (1.79)	0.229

*Baseline comparability of the randomized groups was assessed using independent samples Student’s t-tests for continuous variables meeting the assumption of normality and Mann-Whitney tests for variables violating the normal distribution assumption. Chi-square tests and Fisher’s exact test were run for dichotomous variables. No significant differences between randomized groups were found*.

## Discussion

The study investigates whether a choral singing intervention can prevent or delay cognitive impairment in an older population at increased risk of cognitive decline. Although theoretically plausible, its effectiveness has not been studied scientifically in an RCT. In this study, participants are randomly allocated, with clinical investigators and assessors at each follow-up blinded to the group allocation. The sample size is suitably large and the relatively long intervention period compared to previous singing studies allows for analyzing more long-term effects and increases the chances for the intervention to show efficacy. The use of an active control arm rather than an empty control or “usual care” will distinguish the effect of choral singing from the benefit of activities in a group context. This study thus provides a rigorous test of choral singing’s effectiveness in preventing cognitive decline. Analysing patterns of change over time with a detailed biomarker and MRI assessment battery will allow investigation into potential mechanisms behind the effects of the intervention.

A comprehensive set of measures was designed to encompass the wide range of cognitive, emotional and physical effects of choral singing. However, the many secondary outcome measures taken at multiple time points open the study up to the problem of multiple comparisons problem. Statistical adjustments for the number of comparisons will be made. A multidomain composite cognition score not only reduces the need for multiple comparisons, but is optimal for detecting very early cognitive changes in non-impaired individuals as it is more sensitive to changes than its individual components (Coley et al., 2016; Mormino et al., 2017).

The relative lack of diversity in the sample is a potential limitation. Thus far, participants are predominantly ethnic Chinese and women. Women tend to report stronger well-being effects from choral singing compared to men (Sandgren, 2009), possibly inflating any positive effects of the intervention. The study is also specific to Singaporean older adults, reducing the generalizability of findings. However, the research on choral singing conducted around the world suggests that its benefits transcend culture and countries (Clift and Hancox, 2010).

Given the length of the intervention, attrition will be an issue. Though baseline measures currently show no significant differences between the randomized groups, it is possible that less healthy participants are more likely to drop out of the choral singing arm as the intervention is a more demanding activity than health education. To account for possible bias due to attrition, attendance is tracked. When participants are unable to attend sessions or unable to continue, their reasons will be recorded and monitored over the course of the study.

We aim to produce firm data on the 2-year efficacy of choral singing in preventing cognitive decline. This will form the evidence base for further research and future interventional initiatives, including the scaling up of choral singing as a viable preventive public health program.

## Author Contributions

JT drafted the work and contributed to the design of the study. LF made instrumental contributions to the conception, design and implementation of the study as trial Principal Investigator. He had full access to all data and was responsible for the integrity of data and results. FHMT and BL made substantial contributions to the conception and design of the study. EO was involved in the acqusition of the data and critical revision for intellectual content. KSDL made substantial contributions to the design of the intervention and was involved in its implementation. LGG designed the health education program for the control arm. C-HT, IK-MC, AL, RF, ML, CKYW, JS, JL, RM and E-HK were involved in the design of the study and contributed to the analysis of the results. They also made critical revisions for important intellectual content. All authors provide approval for publication of the content and agree to be accountable for all aspects of the work in ensuring that questions related to the accuracy and integrity of all parts of the work are appropriately investigated and resolved.

## Conflict of Interest Statement

FHMT is the co-founder and co-owner of Maurine Tsakok Inc. The company played no role in conceptualization, design and implementation of the research study. The remaining authors declare that the research was conducted in the absence of any commercial or financial relationships that could be construed as a potential conflict of interest.
