# Supplementary material for: Study Protocol for a Randomized Controlled Trial of Choral Singing Intervention to Prevent Cognitive Decline in At-Risk Older Adults Living in the Community
Source: Front Aging Neurosci. 2018 Jul 10;10:195. doi: 10.3389/fnagi.2018.00195 (PMC6048740; doi:10.3389/fnagi.2018.00195)
Supplement: Supplementary file 1 [file Table_1.docx]

| **Age** |  | **60 to 64 years old** | | |  | **65-69 years old** | | |  | **70 to 74 years old** | | |  | **75 years old & above** | | |
| --- | --- | --- | --- | --- | --- | --- | --- | --- | --- | --- | --- | --- | --- | --- | --- | --- |
| **Education** |  | **0-3 years** | **4-6 years** | **>6 years** |  | **0-3 years** | **4-6 years** | **>6 years** |  | **0-3 years** | **4-6 years** | **>6 years** |  | **0-3 years** | **4-6**  **years** | **>6**  **years** |
| Forward digit span |  | 7.2  (1.7) | 7.4  (1.6) | 7.5  (1.6) |  | 7.3  (1.4) | 7.4  (1.4) | 7.4  (1.5) |  | 7.4  (1.6) | 6.8  (1.6) | 7.0  (1.3) |  | 7.2  (1.6) | 7.2  (1.4) | 7.2  (1.8) |
| Backward digit Span |  | 3.9  (1.4) | 4.3  (1.1) | 4.7  (1.2) |  | 3.5  (0.8) | 4.1  (1.1) | 4.5  (1.1) |  | 3.5  (0.9) | 3.7  (1.3) | 3.8  (0.8) |  | 3.3  (1.1) | 3.8  (1.2) | 4.0  (1.1) |
| RAVLT immediate recall: ∑Tx, x=1..5 |  | 44.0  (8.5) | 42.7  (9.5) | 49.4  (9.2) |  | 39.2  (8.9) | 42.3  (9.0) | 45.0  (9.6) |  | 37.8  (8.2) | 42.3  (8.6) | 39.0  (7.5) |  | 35.6  (10.1) | 35.7  (7.3) | 35.8  (9.9) |
| RAVLT delayed recall |  | 8.8  (3.8) | 8.8  (3.3) | 10.4  (2.7) |  | 8.2  (3.3) | 8.5  (3.5) | 8.7  (3.2) |  | 7.5  (2.9) | 9.2  (2.8) | 8.2  (3.2) |  | 7.2  (3.7) | 6.3  (3.5) | 7.0  (4.5) |
| Colour Trail 1 |  | 93.2  (55.8) | 72.7  (33.4) | 60.5  (23.7) |  | 85.5  (25.6) | 84.4  (38.4) | 68.8  (27.8) |  | 118.8  (52.9) | 82.3  (23.4) | 79.3  (24.8) |  | 100.7  (37.3) | 105.2  (38.0) | 64.0  (18.8) |
| Colour Trail 2 |  | 135.6  (45.3) | 125.7  (37.2) | 110.8  (31.3) |  | 141.0  (32.5) | 147.9  (49.1) | 117.6  (35.0) |  | 169.0  (46.7) | 149.0  (38.1) | 137.7  (37.3) |  | 180.3  (47.3) | 160.4  (44.7) | 120.3  (17.8) |
| Block Design |  | 23.7  (8.5) | 25.5  (8.6) | 32.0  (11.4) |  | 22.3  (8.8) | 24.2  (8.0) | 29.9  (9.3) |  | 20.2  (6.9) | 25.6  (7.8) | 26.6  (9.1) |  | 21.5  (8.1) | 21.4  (4.5) | 29.3  (5.3) |
| Boston Naming Test |  | 19.9  (4.8) | 21.0  (4.8) | 24.3  (4.3) |  | 19.3  (4.1) | 20.9  (3.7) | 24.5  (3.8) |  | 18.8  (3.4) | 20.2  (4.9) | 22.5  (3.7) |  | 16.4  (4.6) | 19.8  (3.6) | 20.7  (3.4) |

Reference:

Lee CK, Collinson SL, Feng L, Ng TP. Clinical Neuropsychologist. 2012; 26(2)321-34

For Colour Trail Test, note that longer time represent poorer performance, remember to add a negative sign to the Z score calculate based on ‘seconds’.
