# Supplementary material for: Study Protocol for a Randomized Controlled Trial of Choral Singing Intervention to Prevent Cognitive Decline in At-Risk Older Adults Living in the Community
Source: Front Aging Neurosci. 2018 Jul 10;10:195. doi: 10.3389/fnagi.2018.00195 (PMC6048740; doi:10.3389/fnagi.2018.00195)
Supplement: Supplementary file 2 [file Table_2.DOCX]

| **Age** |  | **60-69 years old** | |  | **70 years old and above** | |
| --- | --- | --- | --- | --- | --- | --- |
| **Education** |  | **0-6 years** | **>6 years** |  | **0-6 years** | **>6 years** |
| SDMT written |  | 26.3  (9.6) | 42.3  (9.0) |  | 21.3  (10.2) | 31.5  (8.6) |
| SDMT oral |  | 35.0  (10.6) | 49.7  (10.1) |  | 29.3  (10.8) | 39.2  (10.7) |

Unpublished data: Feng L, 20 August 2015; the subjects were 236 SLAS 1 participants with MMSE>=26; assessed at SLAS baseline from 2013 to 2015.
